# Anti-neutrophil cytoplasmic antibody-associated vasculitis accompanied by type II heparin-induced thrombocytopenia resulting in asymptomatic cerebral infarction: a case report

**DOI:** 10.1186/s12882-021-02433-8

**Published:** 2021-06-14

**Authors:** Yoshitaka Furuto, Mariko Kawamura, Jumpei Yamashita, Takahiro Yoshikawa, Akio Namikawa, Rei Isshiki, Hiroko Takahashi, Yuko Shibuya

**Affiliations:** Department of Hypertension and Nephrology, NTT Medical Centre, 5-9-22, Higasi- Gotanda, Shinagawa-ku, 141-8625 Tokyo, Japan

**Keywords:** Anti-neutrophil cytoplasmic antibody-associated vasculitis, Heparin-induced thrombocytopenia, Cerebral infarction, Autoimmune disease, Rapidly progressive glomerulonephritis, Heparin, Nafamostat, Argatroban

## Abstract

**Background:**

Heparin-induced thrombocytopenia (HIT) involves platelet activation and aggregation caused by heparin or HIT antibodies associated with poor survival outcomes. We report a case of HIT that occurred after hemodialysis was started for rapidly progressive glomerulonephritis (RPGN), which was caused by anti-neutrophil cytoplasmic antibody-associated vasculitis (AAV), and ultimately resulted in asymptomatic cerebral infarction.

**Case presentation:**

A 76-year-old Japanese man was urgently admitted to our hospital for weight loss and acute kidney injury (serum creatinine: 12 mg/dL). Hemodialysis therapy was started using heparin for anticoagulation. Blood testing revealed elevated titers of myeloperoxidase anti-neutrophil cytoplasmic antibodies, and renal biopsy revealed crescentic glomerulonephritis with broad hyalinization of most of the glomeruli and a pauci-immune staining pattern. These findings fulfilled the diagnostic criteria for microscopic polyangiitis, and the patient was diagnosed with RPGN caused by AAV. Steroid pulse therapy, intermittent pulse intravenous cyclophosphamide, and oral steroid therapy failed to improve the patient’s renal function, and maintenance dialysis was started. However, on day 15, his platelet count had decreased to 47,000/µL, with clotting observed in the hemodialysis catheter. Magnetic resonance imaging of the head identified acute asymptomatic brain infarction in the left occipital lobe, and a positive HIT antibody test result supported a diagnosis of type II HIT. During hemodialysis, the anticoagulant treatment was changed from heparin to argatroban. Platelet counts subsequently normalized, and the patient was discharged. A negative HIT antibody test result was observed on day 622.

**Conclusions:**

There have been several similar reports of AAV and HIT co-existence. However, this is a rare case report on cerebral infarction with AAV and HIT co-existence. Autoimmune diseases are considered risk factors for HIT, and AAV may overlap with other systemic autoimmune diseases. To confirm the relationship between these two diseases, it is necessary to accumulate more information from future cases with AAV and HIT co-existence. If acute thrombocytopenia and clotting events are observed when heparin is used as an anticoagulant, type II HIT should always be considered in any patient due to its potentially fatal thrombotic complications.

## Background

Heparin is the most commonly used anticoagulant agent during hemodialysis because of its cost-effectiveness. However, heparin-induced thrombocytopenia (HIT) can develop via an immunological response after the start of heparin treatment and reportedly occurs in 3.9 % of patients during the introductory phase of hemodialysis therapy, which frequently causes fatal thrombotic complications [1]. Furthermore, the development of HIT is associated with increased mortality among hemodialysis patients [2].

Cases of HIT can be classified as type I and type II [3]. Type I HIT is related to direct platelet stimulation by heparin, without an immune-related mechanism, leading to a mild decrease in the platelet count at 1–2 days after starting the heparin treatment. However, type I HIT is generally not problematic, as it is not associated with thromboembolic complications, and the platelet counts spontaneously normalize.

Type II HIT is caused by antibodies that recognize the platelet factor 4-heparin complex (HIT antibodies), and it is relatively rare (0.5–5 % of heparin-treated cases) [4]. HIT antibodies cause the activation of platelets, monocytes, vascular endothelial cells, and coagulation factors, resulting in the overproduction of thrombin, and ultimately, thrombopenia and thrombosis [5]. Moreover, patients often develop serious complications that are related to arteriovenous thrombosis and embolism [6]. Anti-neutrophil cytoplasmic antibodies (ANCA) were firstly reported in 1982 as a group of autoantibodies, mainly of the IgG type, against antigens in the cytoplasm of neutrophil granulocytes [7].

Anti-neutrophil cytoplasmic antibody-associated vasculitis (AAV) is a group of relatively rare autoimmune diseases of unknown cause characterized by inflammation of blood vessels and the detection of ANCA in the blood. According to the European League Against Rheumatism [8], AAV has features of inflammation, vasculitis, rapidly progressive glomerulonephritis (RPGN), characteristic renal biopsy findings such as crescent formation, and/or ANCA-positive results. Granulomatosis with polyangiitis (GPA), microscopic polyangiitis (MPA), and eosinophilic granulomatosis with polyangiitis (EGPA) are included in AAV. Herein, we report a rare case of type II HIT that occurred after hemodialysis therapy was started for RPGN, which was caused by AAV, and ultimately resulted in asymptomatic cerebral infarction.

## Case presentation

A 76-year-old Japanese man presented with a chief complaint of general fatigue, as well as weight loss that began in July 2018. He visited a nearby clinic in October 2018 because of his general fatigue and was referred to our hospital because of severe renal dysfunction (serum creatinine: 12 mg/dL). The patient was previously healthy with no notable medical or family history, including no history of allergy. He had smoked 30 cigarettes/day for approximately 55 years and occasionally consumed alcohol.

Physical examination revealed a height of 157 cm, a weight of 44.6 kg, body mass index of 18.1 kg/m^2^, blood pressure of 162/77 mmHg, heart rate of 89 beats/min, and body temperature of 36.6 °C. The patient was lucid and had signs of anemia at the palpebral conjunctiva, but no signs of jaundice at the bulbar conjunctiva. Furthermore, we did not observe any significant findings during examinations of the oral cavity, cervical lymph nodes, cardiopulmonary sounds, abdomen, bilateral costovertebral angle, feet, joints, skin, or related neurological findings.

The blood and urine test results obtained on admission are shown in Table 1. The blood tests revealed severe renal dysfunction, anemia, and elevated levels of C-reactive protein and myeloperoxidase ANCA. The urinalysis revealed positivity for protein and occult blood. Computed tomography revealed evidence of interstitial pneumonia in the posterior segments of the bilateral lower lobes and mild bilateral kidney enlargement, suggesting acute kidney injury. Based on these findings, the patient was urgently admitted for treatment.

**Table 1:** Laboratory findings from the admission

Urinalysis		Biochemistry, immunological, virologic, and coagulation parameters			
Protein	3+	TP	6.9 g/dL	Ferritin	499 ng/mL
Occult blood	3+	Alb	3.3 g/dL	IgG	1,674 mg/dL
Red blood cells	> 100/HPF	UA	8.3 mg/dL	IgA	296 mg/dL
Protein	5.72 g/gCr	BUN	134.2 mg/dL	IgM	69 mg/dL
NGAL	1485 ng/mL	Cr	13.96 mg/dL	C3	79 mg/dL
NAG	17.8 U/L	eGFR	3 mL/min/1.73 m^2^	C4	27.3 mg/dL
α1MG	91.8 mg/L	TB	0.1 mg/dL	CH50	43 U/mL
β2MG	37,676 µg/L	AST	6 IU/L	Antinuclear Ab	1:40
Bence Jones protein	–	ALT	10 IU/L	Anti-dsDNA Ab	–
**Complete blood cell count**		ALP	249 IU/L	Anti-smith Ab	–
White blood cells	6,300/µL	γ-GT	16 IU/L	Anti-CL-β2GP1 Ab	–
Neutrophils	75.10 %	LDH	166 IU/L	Direct Coombs test	–
Lymphocytes	13.30 %	CK	64 IU/L	Anti-MPO-ANCA	1,700 IU/mL
Monocytes	5.90 %	Na	134 mEq/L	Anti-PR3-ANCA	–
Eosinophils	5.20 %	K	6.3 mEq/L	Cryoglobulin	–
Red blood cells	238 × 10^4^/µL	Cl	102 mEq/L	HIV Ag/Ab	–
Hemoglobin	7.5 g/dL	cCa	7.7 mg/dL	HBs Ag/HBc Ab	–
Hematocrit	22.60 %	IP	7.3 mg/dL	HCV Ab	–
Platelets	15.8 × 10^4^/µL	CRP	2.47 mg/dL	PT	94 %
		TC	147 mg/dL	APTT	24.9 s
		TG	220 mg/dL	D-dimer	4.1 µg/mL
		HbA1c	5.40 %		
		HANP	340.6 pg/mL		
		BNP	140.0 pg/mL		

*NAG* N-acetyl-beta-D-glucosaminidase, *NAGL* NAG ligand, *α1MG* alpha1-microglobulin, *β2MG* beta2-microglobulin, *TP* total protein, *Alb* albumin, *UA* uric acid, *BUN* blood urea nitrogen, *Cr* creatinine, *eGFR* estimated glomerular filtration rate, *AST* aspartate transaminase, *ALT* alanine transaminase, *ALP* alkaline phosphatase, *γ-GT* gamma-glutamyl transferase, *LDH* lactate dehydrogenase, *CK* creatine kinase, *CRP* C-reactive protein, *TC* total cholesterol, *TG* triglycerides, *HBa1c* glycated hemoglobin, *BNP* brain natriuretic peptide, *ANCA* anti-neutrophil cytoplasmic autoantibody, *HIV* human immunodeficiency virus, *PT* prothrombin time, *APTT* activated partial thromboplastin time

**Fig. 1:**
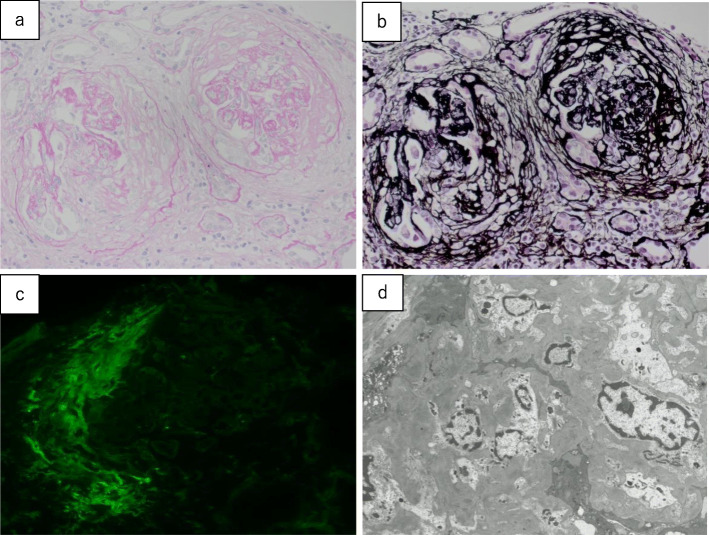
Renal biopsy findings. **a** Periodic acid-Schiff staining (40×), **b** periodic acid-methenamine silver staining (40×), **c** immunostaining for fibrinogen, and **d **electron microscopy findings (2,000×). Fifteen glomeruli (3 were completely hyalinized, and 12 were mostly hyalinized) exhibit evident crescent formation, with transitioning to fibrous crescents. The glomerular loops are collapsed and destroyed with fibrin deposition. Expansion of the mesangial matrix and an increase in the mesangial cell count are observed, although no double contours or spike formation of the glomerular basement membranes are observed. Lymphocyte infiltration is evident in the tubular interstitium, and 60–70 % of all renal tubules are atrophied. Moderate arteriosclerosis resulting from intimal thickening and medial atrophy is observed in the interlobular arteries (**a, b**). Immunofluorescence failed to detect the expression of IgG, IgA, IgM, C3, C4, or C1q, and only fibrinogen expression is observed in the crescents (**c**). Electron microscopy reveals no electron-dense deposits, and podocyte degeneration is observed with evident disappearance of the podocyte foot processes (**d**)

Hemodialysis was started for acute kidney injury on day 1 using heparin as an anticoagulant. Renal biopsy was performed on day 7, which revealed that 3 of 15 glomeruli were completely hyalinized, and the remaining 12 glomeruli were mostly hyalinized, with crescent formation and transitioning to fibrous crescents, as well as collapsed and destroyed glomerular loops (Fig. 1). Lymphocyte infiltration was observed in the tubulointerstitium, and 60–70 % of all renal tubules were atrophied. Immunostaining revealed a pauci-immune pattern, and these findings fulfilled the diagnostic criteria for MPA. Thus, the patient was diagnosed with RPGN related to acute kidney injury caused by AAV.

**Fig. 2:**
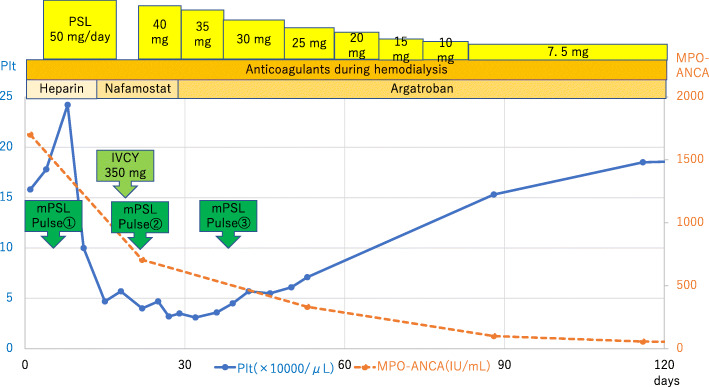
The treatment course and changes in platelet count over time. The treatments involved steroid therapy with prednisolone (PSL), anticoagulant treatment during hemodialysis, intravenous cyclophosphamide (IVCY) therapy, and steroid pulse therapy with methylprednisolone (mPSL). The laboratory test parameters were changes in myeloperoxidase anti-neutrophil cytoplasmic antibody (MPO-ANCA) titers (red) and platelet counts (blue). The platelet counts improved after argatroban was started as anticoagulant treatment during hemodialysis

The Birmingham Vasculitis Activity Score was 18, and no alveolar hemorrhage or neurological symptoms were observed. The patient was treated using three sessions of steroid pulse therapy (methylprednisolone at 1,000 mg/day), one session of intermittent intravenous cyclophosphamide pulse (IVCY) therapy (because we considered avoiding aggressive immunosuppression since the kidney biopsy results showed more chronic changes and fibrosis/atrophy than the active disease), and oral steroid therapy (prednisolone at 50 mg/day) (Fig. 2). However, the patient’s renal function did not improve, and maintenance dialysis was started.

On day 15, clotting was observed in the hemodialysis catheter. Complete blood count with biochemical parameters was performed as a follow-up to assess anemia and the state of AAV after steroid pulse therapy revealed a drastic drop in the patient’s platelet count to 47,000/µL without elevated levels of C-reactive protein. HIT related to hemodialysis using heparin was strongly suspected, and D-Dimer with other coagulation parameters were evaluated to assess diseases causing thrombocytopenia. The D-dimer concentration had increased acutely to 22.7 µg/mL up from 4.1 µg/mL on admission despite the negative level of C-reactive protein after AAV treatment suggesting improvement of AAV. Because type II HIT can potentially cause critical thrombosis, physical and image examinations were performed based on a suspicion of thrombosis.

The patient was lucid after hemodialysis initiation, symptoms were absent, and vital signs were as follows: blood pressure of 133/68 mmHg, heart rate of 67 beats/min, body temperature of 36.2 °C, and oxygen saturation of 99 %. Regarding physical findings, no findings of vision loss, restricted visual field, abnormal cardiopulmonary sounds, abdominal pain, and livedo reticularis were observed. The pulse of the bilateral radial arteries and the bilateral dorsalis pedis artery were normal. There were no findings of motor paralysis, sensory disorders, gait disturbance, dysarthria, aphasia, and unilateral spatial neglect. No significant neurological findings were observed, and no findings of thromboembolism were observed in the trunk or limbs. However, we performed head magnetic resonance imaging and computed tomography of the trunk to establish differential diagnoses of thrombosis, inflammatory disease, and chronic diseases related to acute thrombocytopenia with an increase in D-dimer concentration and clotting in the hemodialysis catheter. This is because we consider that AAV is often accompanied by thrombosis and is rarely accompanied by cerebral aneurysm or arterial stenosis resulting from vasculitis. Moreover, thrombosis could similarly be caused by type II HIT.

**Fig. 3:**
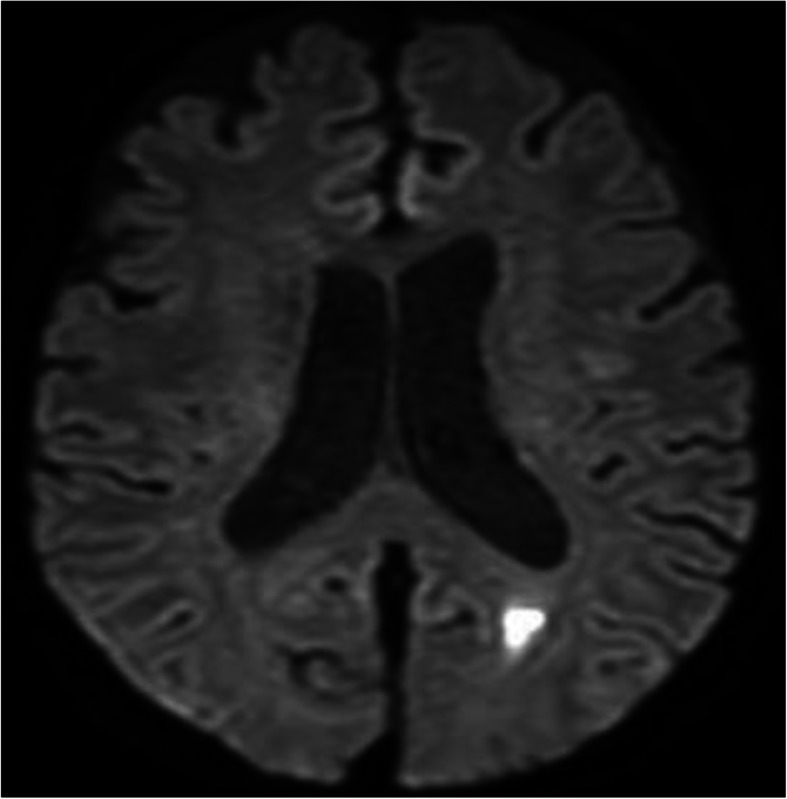
Diffusion-weighted magnetic resonance imaging of the head reveals a high-intensity signal in the left occipital-temporal lobe, which indicates acute cerebral infarction

Consequently, acute asymptomatic cerebral infarction was observed in the left occipital lobe without findings of vasculitis in cerebral arteries during head magnetic resonance imaging (Fig. 3). In contrast, computed tomography of the trunk revealed no findings of thrombosis, inflammatory disease, and chronic diseases. Thus, HIT was suspected based on the decreased platelet count after starting heparin treatment and the development of asymptomatic cerebral infarction, as well as clotting in the hemodialysis catheter. Disseminated intravascular coagulation was considered unlikely based on the absence of infection or malignant tumors, and thrombotic microangiopathy was considered unlikely based on the haptoglobin value being within the normal range. Furthermore, idiopathic thrombocytopenic purpura was considered unlikely based on the negative results for anti-platelet antibodies (Table 2), and there was no atrial fibrillation. However, a high likelihood of HIT was judged based on the HIT scoring system (a 4Ts score of 6) [9], and the presence of HIT antibodies on day 15 supported a diagnosis of asymptomatic cerebral infarction caused by type II HIT (Table 2). Because there was no bleeding lesion in the brain, the patient was treated using aspirin (200 mg/day).

**Table 2:** Laboratory findings from day 15

Platelets	4.7 × 10^4^/µL
CRP	< 0.3 mg/dL
D-dimer	22.7 µg/mL
Haptoglobin	160 mg/dL
Anti-thrombocyte antibodies	−
Anti-platelet factor 4-heparin complex antibodies	+, 5.6 U/mL

*CRP* C-reactive protein

After thrombocytopenia was confirmed, the anticoagulant treatment during hemodialysis was changed from heparin to nafamostat on day 15, which we have commonly used for patients in whom heparin is contraindicated. However, the platelet count did not begin to recover until after the anticoagulant treatment was changed from nafamostat to argatroban on day 29. The platelet counts gradually improved, and the patient was discharged on day 51, with argatroban treatment during the subsequent hemodialysis. The patient did not experience any vascular events after the discharge, and a negative result was observed for a second HIT antibody test that was performed on day 622.

## Discussion and conclusions

This report describes our experience with a case of type II HIT that occurred after hemodialysis therapy was started for RPGN, which was caused by AAV, and ultimately resulted in asymptomatic cerebral infarction. Asymptomatic cerebral infarction was probably caused by either HIT or AAV, or a combination of both, because they both evidently induce arteriolar infarction. However, because this is diagnosed as acute cerebral infarction with diffusion-weighted magnetic resonance imaging of the head without findings of vasculitis in the cerebral arteries and with negative levels of C-reactive protein after AAV treatment suggesting improvement of AAV, we consider that this asymptomatic cerebral infarction was more likely caused by type II HIT than by AAV.

The principal stroke mechanism of posterior cerebral artery (PCA) infarction was suggested to be cardioembolic (54.1 %) in the superficial PCA territory, lacunar (46.2 %) in the proximal PCA territory, and undetermined (40.2 %) in both the proximal and the superficial territories; thus, we believe that the mechanism underlying cerebral infarction in this case is “undetermined” [10]. The patient’s platelet count improved after heparin was replaced with argatroban as the anticoagulant. In this context, type II HIT is caused by antibodies that bind to the platelet factor 4-heparin complex and form an immune complex, which leads to platelet activation and a hypercoagulable state that causes arterial and venous thrombosis [9, 11]. The emergence of HIT typically occurs 5–14 days after the start of heparin treatment, and thrombocytopenia usually resolves 7–10 days after the discontinuation of heparin treatment. Clinical suspicion of HIT is based on a sharp decrease in platelet counts during heparin treatment, which generally resolves after the heparin is discontinued. The diagnosis of HIT is based on a clinical scoring system [12, 13] and serologically supported by the detection of HIT antibodies [14]. The differential diagnoses of HIT include other pathological conditions that cause thrombocytopenia during the acute phase of AAV, such as thrombotic microangiopathy [15], non-heparin drug-induced thrombocytopenia, and disseminated intravascular coagulation caused by infection.

The treatment of HIT involves complete cessation of heparin treatment during hemodialysis, with a switch to argatroban (an antithrombin drug) [9, 16]. Nafamostat may be an alternative anticoagulant agent during hemodialysis; however, it cannot suppress the hypercoagulable state induced during the acute phase of HIT and does not help improve platelet counts. Therefore, in Japan, argatroban is a more desirable and established anticoagulant agent for use during the acute phase of HIT. However, HIT antibodies only exist temporarily and reportedly become undetectable at 50–85 days after the discontinuation of heparin [17]. When the HIT antibodies become undetectable, nafamostat and argatroban can be used as anticoagulant agents during maintenance dialysis. In addition, re-administering heparin or low-molecular-weight heparin while monitoring for HIT antibodies did not cause HIT recurrence [18–20]. Although immunosuppressive therapy is rarely used for HIT, there was one reported case of refractory HIT treated using plasma exchange and rituximab [21]. High-dose intravenous immunoglobulin may also be an effective adjunct to anticoagulation treatment for HIT [22].

Although HIT and AAV have different etiologies, HIT is thought to involve autoimmune mechanisms, as its pathophysiology is related to the production of immune complexes. Titers of HIT antibodies (IgM, IgA, and IgG) increase simultaneously from the fifth day after the start of heparin treatment [23], and HIT IgG antibodies activate platelets, monocytes, and vascular endothelial cells, leading to the development of HIT [24]. Thus, HIT may involve a mechanism that is different from a normal antigen-antibody reaction. Furthermore, HIT is significantly more common among patients undergoing hemodialysis and those with autoimmune diseases, as autoimmune diseases are reportedly a risk factor for HIT because of abnormal immune complex production [25]. Moreover, there are statistically significant associations between the prevalence of HIT and several autoimmune diseases, including antiphospholipid syndrome, systemic lupus erythematosus, rheumatoid arthritis, Hashimoto’s thyroiditis, and nonischemic cardiomyopathy [26]. Thus, although the association between HIT and autoimmune disease is not clearly understood, the development of an autoimmune disease and HIT may have some commonalities. First, HIT requires the formation of a specific “neoantigen” (the heparin-platelet factor 4 complex), which is similar to the citrullinated proteins that are central to the pathogenesis of rheumatoid arthritis [27, 28]. Second, IgG antibodies that bind this complex can be detected for some period of time, similar to the post-vaccination Arthus reaction (type III hypersensitivity reaction) [29]. Because the most frequent autoimmune disease associated with AAV is rheumatoid arthritis, followed by the other systemic autoimmune diseases [30], AAV may be related to these mechanisms. Although the co-occurrence of AAV and HT is rare, a search of the PubMed database revealed several reports of HIT that occurred at the start of hemodialysis therapy for AAV, which is similar to that in the present case [31–35] (Table 3). Regarding the clinical characteristics of the six cases of coexistent AAV and HIT, including this case, all cases involved immunosuppressive therapy and used unfractionated heparin for dialysis or prevention of deep vein thrombosis. The onset of HIT from heparin use was 10.7 ± 3.6 days. Five of six patients had thrombotic or clotting events, and five of six patients were treated with argatroban for HIT. However, the present case is a rare case report of asymptomatic cerebral infarction with AAV and HIT co-existence.

**Table 3:** Clinical characteristics of six cases of coexisting AAV and HIT, including this case

Literature	Age	Sex	Type of ANCA	Organ injury	Treatment for AAV	Onset of HIT	Type of heparin	Thrombotic event	Treatment for HIT
**Roe et al.** [31].	65	M	PR3	Crescentic glomerulonephritis Pulmonary hemorrhage	mPSL pulse, PSL, CY Hemodialysis	9 days after the start of hemodialysis	Unfractionated heparin	Deep venous thrombosis	Danaparoid
**Kaneda et al.** [32].	91	F	MPO	Kidney dysfunction Pulmonary hemorrhage	mPSL pulse, PSL, Hemodialysis	13 days after the start of hemodialysis	Unfractionated heparin	N/A	Argatroban
**Mandai et al.** [33].	40	M	MPO	Crescentic glomerulonephritis Interstitial pneumonia	mPSL pulse, PSL, CY, PE, Hemodialysis	5 days after the start of hemodialysis	Unfractionated heparin	Clotting in hemodialysis catheter	Argatroban
**Thong et al.** [34].	71	M	PR3	Kidney dysfunction	mPSL pulse, PSL, CY, Hemodialysis	15 days after the start of hemodialysis	Unfractionated heparin	Circuit and catheter clotted	Argatroban
**Nonaka et al.** [35].	87	F	MPO	Kidney dysfunction	PSL Hemodialysis	8 days after the start for prevention of deep vein thrombosis	Unfractionated heparin	Deep venous thrombosis	Argatroban
**This case**	76	M	MPO	Crescentic glomerulonephritis Interstitial pneumonia	mPSL pulse, PSL, CY Hemodialysis	14 days after the start of hemodialysis	Unfractionated heparin	cerebral infarction Clotting in hemodialysis catheter	Aargatroban

*ANCA* Anti-neutrophil cytoplasmic antibody, *AAV* anti-neutrophil cytoplasmic antibody-associated vasculitis, *HIT* heparin-induced thrombocytopenia, *PR3* proteinase 3, *MPO* myeloperoxidase, *mPSL *methylprednisolone, *PSL* prednisolone, *CY *cyclophosphamide, *PE *plasma exchange

Most patients in these reported cases had received immunosuppressive therapy, but only developed HIT after starting hemodialysis for AAV-induced renal failure. Therefore, the immunosuppressive therapy may not suppress the production of antibodies that target the platelet factor 4-heparin complex. However, no reports have examined the relationship between AAV and HIT, and the precise reason for the co-existence of these two diseases is poorly understood. Nevertheless, HIT causes thromboembolic diseases in approximately 20–50 % of patients [6] and has a mortality rate of approximately 5 % [36]. Moreover, AAV is a significant risk factor for deep vein thrombosis [9, 37, 38].

In conclusion, there are several reports regarding the co-existence of AAV and HIT. To confirm the relationship between these two diseases, it is necessary to conduct further studies on cases with AAV and HIT co-existence. If acute thrombocytopenia and clotting events are observed when heparin is used as an anticoagulant, type II HIT should always be considered in any patient due to its potentially fatal thrombotic complications.

## Data Availability

Not applicable.

## Abbreviations

AAV: Anti-neutrophil cytoplasmic antibody-associated vasculitis
ANCA: Anti-neutrophil cytoplasmic antibodies
HIT: Heparin-induced thrombocytopenia
PCA: Posterior cerebral artery
RPGN: Rapidly progressive glomerulonephritis
